# Louse-borne relapsing fever – report of four cases in Switzerland, June-December 2015

**DOI:** 10.1186/s12879-016-1541-z

**Published:** 2016-05-17

**Authors:** Michael Osthoff, Adrian Schibli, Davide Fadini, Pietro Lardelli, Daniel Goldenberger

**Affiliations:** Division of Infectious Diseases & Hospital Epidemiology, University Hospital Basel, Petersgraben 4, Basel, 4031 Switzerland; Division of Infectious Diseases & Hospital Epidemiology, Triemli Hospital, Birmensdorferstrasse 497, Zurich, 8063 Switzerland; Emergency Service, Ospedale Regionale, Via Turconi 23, Mendrisio, 6850 Switzerland; Department of Internal Medicine, Ospedale Regionale, Via Turconi 23, Mendrisio, 6850 Switzerland; Division of Clinical Microbiology, University Hospital Basel, Petersgraben 4, Basel, 4031 Switzerland

**Keywords:** Borrelia recurrentis, Emerging diseases, Broad-range bacterial PCR, Relapsing fever, Migrants

## Abstract

**Background:**

Louse-borne relapsing fever (LBRF) is a neglected disease that has been restricted to East Africa for many decades. Several cases in refugees from the Horn of Africa have been recently diagnosed in four European countries.

**Case presentation:**

We report four additional cases of LBRF in asylum seekers from Somalia and Eritrea who presented with fever shortly after arriving in Switzerland during a seven-month period. Multiple spirochetes were visualized on stained blood films which were identified as *Borrelia recurrentis* by 16S rRNA gene sequencing. All patients recovered after antibiotic treatment with ceftriaxone and/or doxycycline. Concurrent infections (malaria and tuberculosis) were diagnosed in half of our patients. Possible modes of transmission and preventive measures are discussed.

**Conclusions:**

These reported cases highlight the ongoing transmission of LBRF in migrants from East Africa. Diagnosis of LBRF cases and prevention of autochthonous transmission in asylum seeker camps are important steps for the near future.

## Background

Once a major epidemic disease in Africa and Eurasia, louse-borne relapsing fever (LBRF) caused by *Borrelia recurrentis* has been restricted to East Africa for many decades with most cases reported from Ethiopia [1, 2]. This neglected disease is exclusively transmitted from human to human via the human body louse *Pediculus humanus humanus*, which is linked to areas of overcrowding, war, and destitution [3]. Due to its restricted geographical occurrence und non-specific presentation with fever and body pain resembling other serious infections such as malaria, viral hemorrhagic fever, leptospirosis or typhoid fever, the diagnosis of LBRF might be missed in non-endemic countries. Of note, mortality in untreated patients may be substantial (up to 70 %) [1]. Diagnosis is established by demonstration of spirochetes in stained blood films during febrile episodes, and polymerase chain reaction (PCR) targeting the 16S rRNA gene may be used for confirmation [4]. Treatment of choice is a single dose of procaine penicillin, tetracycline or doxycycline, and patients should be closely monitored for a potentially severe Jarisch-Herxheimer reaction [3, 5].

In the context of an ongoing influx of refugees from East Africa into Europe, more than twenty cases of LBRF in asylum seekers originating from this area have been recently reported from several European countries [6–9] including the first case diagnosed in Switzerland [4] at one of our institutions. Herein, we report four additional, independent cases of LBRF in asylum seekers from the Horn of Africa at our three institutions during a five month period. All participants gave written informed consent for publication of their details and images.

## Case presentation

**Fig. 1:**
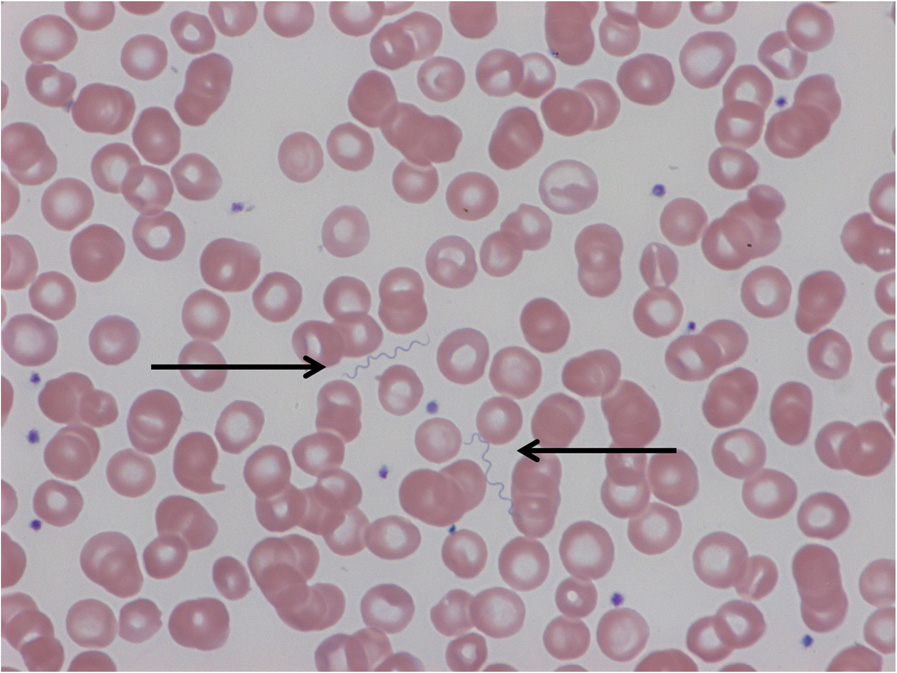
Microscopic detection of spirochetes (black arrows) in blood from patient 2. May-Grünwald Giemsa (MGG)-stained blood smear, 1000-fold magnification

LBRF patients originated from Eritrea and Somalia, respectively, with a final common travel route via Sudan, Libya and Italy before arriving in Switzerland in June to December 2015. All patients were referred to the emergency department of our three institutions with fever and other non-specific complaints shortly (one to seven days) after arrival. Interestingly, all patients reported a previous febrile episode in Italy or Libya. Important clinical characteristics of the patients are shown in the Table 1. All patients denied a significant past medical history or past/current medical treatment. With the exception of enlarged cervical and axillary lymph nodes in patient 3, physical examination was essentially non-specific and consistent with sepsis of unknown origin in the context of fever, tachycardia and hypotension. Routine blood tests were significant for mild to moderate thrombocytopenia, mild anaemia and markedly elevated inflammatory markers in all cases (Table 1). In addition, one patient showed evidence of haemolysis with an increased LDH and bilirubin. Differential diagnosis entertained by the treating clinicians included bacterial sepsis of unknown origin, typhoid fever and malaria in all patients, and primary EBV infection in patient 3. Two sets of blood cultures, stained blood smears and a malaria rapid diagnostic test were ordered in all patients. Diagnosis of *Borrelia*-associated relapsing fever was established by demonstration of abundant extracellular spirochetes in stained blood films (Fig. 1). Additional work-up including blood cultures and malaria tests was negative in patients 1 and 3, and positive for *Mycobacterium tuberculosis* lymphadenitis (lymph node biopsy demonstrating acid fast bacilli on auramine staining and isolation of *M. tuberculosis* from culture) and *Plasmodium falciparum* malaria (*P. falciparum* parasites were observed concomitantly with spirochetes in stained blood films) in patients 2 and 4, respectively. The first two patients were admitted to the intensive-care unit for supportive treatment of sepsis-related hypotension and were started on intravenous ceftriaxone, whereas the last two patients received doxycycline (in addition to standard first-line anti-tuberculous treatment in patient 2 and artemether/lumefantrine in patient 4 for concurrent *Plasmodium falciparum* malaria) and were admitted to the general medical ward after fluid resuscitation. All patients rapidly recovered after antibiotic treatment. Treatment duration was longer than necessary for LBRF to cover for potential tick-borne relapsing fever, as confirmation of LBRF by PCR was not available immediately. A Jarisch-Herxheimer reaction including aggravated hypotension, tachycardia and high-grade fever was only observed in patient 2.

**Table 1:** Characteristics of four patients suffering from louse-borne relapsing fever (*Borrelia recurrentis*) diagnosed in Switzerland from June to December 2015

Case	Sex, age	Origin	Travel route	Location of first febrile episode	Symptoms	Platelet count (on admission, nadir; × 10^9^)	CRP (on admission, peak; mg/L)	Other laboratory features	Treatment
1	M, 25 y	Eritrea	ER, SU, LB, IT, CH	Libya	Fever, abdominal pain, epistaxis	56, 30	312, 312	Mild anemia, hyponatremia and hypokalemia	Ceftriaxone for 2 days followed by doxycycline for 7 days
2	M, 29 y	Somalia	SO, KE, SU, LB, IT, CH	Italy (infestation with body lice in Libya)	Fever, abdominal pain, headache, muscle aches	141, 54	245, 245	Mild anemia, leukocytosis and hyponatremia	Ceftriaxone for 1 day followed by doxycycline for 7 days plus HRZE^b^
3	F, 21 y	Somalia	SO, KE, UG, SU, LB, IT, CH	Italy	Fever, pollakisuria, swollen/painful cervical/axillary lymphadenopathy	45, 27	367, 367	Mild anemia, leukocytosis and hyponatremia, elevated creatinine (mild), bilirubin and LDH (both moderate)	Doxycycline for 5 days
4	M, 17y	Somalia	SO, ET, SU, LB, IT, CH	Libya	Fever, chills, cough, urinary incontinence	167, 62	127, 235	Moderate anemia, mild hyponatremia, hypokalemia, elevated creatinine and LDH (both mild)	Doxycycline for 3 days plus Artemether/lumefantrine^a^

*Abbreviations*: *F* female, *M* male, *Y* year, *CRP* C-reactive protein, *HRZE* antimycobacterial treatment with isoniazid (H), rifampicin (R), pyrazinamide (Z) and ethambutol (E)

Travel route: *CH* Switzerland, *ER* Eritrea, *ET* Ethiopia, *IT* Italy, *LB* Libya, *SU* Sudan, *SO* Somalia, *UG* Uganda

Platelet count, norm: 150–450 × 10^9^/L; C-reactive protein, norm: <10 mg/L

^a^Artemether/lumefantrine treatment was administered for concurrent *Plasmodium falciparum* malaria infection

^b^Antimycobacterial treatment consisting of HRZE for two months followed by HR for four months was administered for concurrent *M. tuberculosis* lymphadenitis

Subsequently, diagnosis of LBRF was confirmed in all cases by broad-range bacterial PCR followed by sequencing of the first half of the 16S rRNA gene using EDTA blood specimens that were positive for spirochetes on microscopy (100 % identity to *B. recurrentis* reference sequence). Based on this result we analysed the entire 16S rRNA gene in two patients, which yielded 1475- (patient 1) and 1480-bp-long (patient 3) sequences (GenBank accession no. KT221542 and KU308247), respectively, with 100 % identity each to *B. recurrentis* reference strain A1 (using the Basic Local Alignment Search Tool (BLAST), National Library of Medicine) [10].

LBRF was once a major epidemic disease in many parts of the world [1]. Lack of an animal reservoir, exclusive transmission of the infectious agent *B. recurrentis* via the human body louse and the association of the latter with poor hygienic conditions during war and destitution are obvious explanations for the fact that LBRF has been rarely encountered in Europe since World War II.

Interestingly, this almost forgotten disease has re-emerged in Europe in the context of the ongoing migration from East Africa. Since July 2015, 26 cases of LBRF in migrants from the Horn of Africa have been reported from four European countries including the Netherlands [9] (two refugees from Eritrea), Germany (15 refugees from Somalia (12), Eritrea (2) and Ethiopia (1)) [8], Italy (eight refugees from Somalia) [6, 7] and Switzerland (one refugee from Eritrea) [4]. Herein, we report four additional cases of LBRF in refugees from Eritrea and Somalia. As symptoms of LBRF are non-specific and a blood-smear is required to diagnose LBRF, these cases may only represent the tip of the iceberg and further cases are expected. The fact that our last patient was only diagnosed in December 2015 confirms ongoing transmission of LBRF in migrants from East Africa and underscores the requirement for continuous vigilance and surveillance for this disease in the future months or even years.

Given the long duration of travel and the short incubation period (2–15 days), infection with *B. recurrentis* did most likely not occur in the country of origin, but along migration routes. All our patients reported a previous febrile episode while travelling through Libya or Italy, which might or might not have been related to LBRF, and one patient even remembered an infestation with body lice during a prison stay in Libya. While most previously reported cases travelled via Sudan and Libya to Italy, the exact mode and location of transmission remains to be determined, but might include spread of Ethiopian foci of LBRF infection into neighbouring countries such as Sudan or introduction of LBRF into camps and shelters in Sudan, Libya or even Italy by migrants after transit of LBRF-endemic Ethiopia. Joint travel of refugees with body lice infested migrants in confined spaces on boats across the Mediterranean Sea might facilitate the spread of LBRF to camps in Europe. This hypothesis is underscored by a recent Italian report of LBRF in two Somalian refugees, who had not travelled outside of Italy for several years, but had resided in the same facility as three recently arrived asylum seekers who were diagnosed with LBRF during the same period of time (July-September 2015) [7]. Hence, introduction and autochthonous transmission of infected body lice and consequently LBRF in asylum seeker facilities in North African or European transit countries seem possible and may pose a challenge for the near future. Nevertheless, it is intriguing, that LBRF infections have not yet been reported in asylum seekers from other parts of Africa, although migration routes partially overlap and often converge in Sudan, Libya or Italy, or from the Middle East (including refugees from Syria). In conclusion, transmission of body lice infected with *B. recurrentis* along migration routes, in particular in Libya, during the passage of the Mediterranean Sea and in Italy seems most likely, in our opinion, but future epidemiological studies are necessary to determine the exact location of transmission.

Given the ongoing migration of asylum seekers from East Africa into Europe, physicians should be vigilant and consider the possibility of LBRF, other louse-transmitted diseases such as epidemic typhus and trench fever, and non-louse-transmitted diseases such as malaria, tuberculosis and typhoid fever when assessing febrile migrants [3]. Concomitant serious, non-louse-transmitted diseases were diagnosed in half of our patients.

As diagnosis of LBRF is established by demonstration of spirochetes in stained blood films during febrile episodes, haematologists need to be aware of this “forgotten” disease. Immediate treatment with procaine penicillin, an intravenous cephalosporin or an oral tetracycline is mandatory. In addition, infection control and prevention measures are crucial to prevent lice transmission in asylum seeker camps in North Africa and Europe. These may include hot washing (60 °C) or preferably exchange of clothes in addition to screening for lice infestation at arrival and preventive delousing of individuals living in close proximity with a case. Complete exchange of clothes is particularly effective, as body lice live in garments (and not on the human skin) and do not survive unless they feed on the blood of human beings every single day [3]. The European Centre for Disease Prevention and Control has issued guidance for infection control measures that should be implemented in the case of LBRF diagnosis [11].

## Conclusions

The future will tell if the almost forgotten disease LBRF will spread to other parts of Africa and Europe in the context of the ongoing migration including refugees from the Middle East. Diagnosis of LBRF cases and prevention of autochthonous transmission in asylum seeker camps are crucial steps for the near future.

### Ethics and consent to participate

Not applicable.

### Consent to publish

All participants gave written informed consent for publication of their details and images. Approval of an ethics committee was not required for this study.

### Availability of data and materials

All data supporting the presented case report is contained within the manuscript.

## Abbreviations

LBRF: louse-borne relapsing fever
PCR: polymerase chain reaction
